# A multi-dataset single-cell meta-analysis of human keloid skin across Asian, Black and White populations reveals endothelial and mesenchymal programs linked to ethnic disparities

**DOI:** 10.3389/fmed.2026.1751725

**Published:** 2026-07-17

**Authors:** Ziad Alkouz, Lian Zhang, Ala'a Al Suwait, Rehab Alhejairi, Chenmei Liu, Xiangyu Gu, Bin Yang

**Affiliations:** Dermatology Hospital of Southern Medical University, Guangzhou, Guangdong, China

**Keywords:** endothelial heterogeneity, ethnicity, fibroblast states, keloid, meta-analysis, scCODA, scVI, single-cell RNA sequencing

## Abstract

**Introduction:**

Keloid is a fibroproliferative scar with marked ethnic disparities, disproportionately affecting Asian and Black populations. Single-cell RNA sequencing (scRNA-seq) studies have mapped keloid pathology, but cross-study differences complicate generalization. We performed a meta-analysis to identify composition and transcriptional programs associated with keloid across ethnicities.

**Methods:**

We aggregated human skin scRNA-seq datasets containing keloid and healthy samples with documented ethnicity (Asian, Black, White). After quality control and batch integration (scVI/scANVI), we annotated cell types and subtypes. Differential abundance was estimated with scCODA, Milo, and propeller. Pseudobulk differential expression was meta-analyzed using random-effects models. Ligand-receptor signaling was inferred with CellChat/NicheNet.

**Results:**

The harmonized atlas comprised 112 donors, 147 samples, and 487,293 cells from 8 studies. Keloid demonstrated higher proportions of vascular endothelial cells and fibroblasts across all methods. Ethnicity-stratified analysis revealed endothelial expansion in Asian and Black relative to White populations, while fibroblast expansion was greater in White keloids. Endothelial subtyping showed increased post-capillary venules and arteriolar states. Fibroblast analysis revealed expansion of mesenchymal/ADAM12^+^ and pro-inflammatory populations. Meta-analysis implicated TGF-*β*/SMAD, integrin/FAK, and YAP/TAZ mechanotransduction pathways as candidate pathogenic programs warranting functional validation.

**Conclusion:**

Cross-study synthesis identifies shared and ethnicity-stratified endothelial and fibroblast programs in keloid. Expansion of post-capillary venules and ADAM12^+^ fibroblasts provides candidate therapeutic targets pending functional validation and a framework for understanding ethnic disparities in keloid formation.

## 1 Introduction

Keloid is a chronic fibroproliferative scarring disorder characterized by exuberant collagen deposition, persistent inflammation, and a propensity to extend beyond wound margins (1–3). The condition represents a significant global health burden with profound psychosocial impacts, particularly affecting quality of life through pruritus, pain, and cosmetic disfigurement (4). Epidemiologically, keloid affects individuals of all backgrounds but demonstrates a striking ethnic disparity: incidence rates reach 16% in Black populations and 3–7% in Asian populations, compared with 0.09% in White populations (5–7). This disparity suggests underlying biological and environmental modifiers that remain incompletely understood.

At the tissue level, keloid pathogenesis involves dysregulated wound healing sustained by reciprocal crosstalk among fibroblasts, endothelial cells, immune populations, and neural elements (8–10). Historical bulk transcriptomic studies identified aberrant TGF-*β* signaling, extracellular matrix (ECM) overproduction, and altered inflammatory programs (11–13). However, these approaches masked cellular heterogeneity and could not resolve cell type-specific contributions to pathogenesis.

Recent single-cell RNA sequencing (scRNA-seq) studies have begun to map cellular diversity within keloid lesions at unprecedented resolution (14–20). Key findings include expansion of mesenchymal/ADAM12^+^ fibroblast subpopulations (14, 21), endothelial activation and angiogenic programs (15, 16), altered immune states including M2-polarized macrophages (17), and neural remodeling (18). However, these individual studies employed divergent analytical pipelines, sequencing platforms (10X Genomics, Smart-seq2, Drop-seq), tissue procurement sites (earlobe, chest, shoulder), and lesion stages (early vs. mature), hindering cross-study generalization and identification of conserved pathogenic mechanisms.

Furthermore, despite the well-documented ethnic disparities in keloid incidence and severity, no study has systematically compared single-cell profiles across ethnic groups. Understanding ethnicity-specific cellular and molecular programs could inform precision medicine approaches and address health disparities in keloid treatment.

We hypothesized that a rigorous, study-aware meta-analysis of scRNA-seq datasets would: (i) identify cell type/subtype composition shifts consistently associated with keloid across studies; (ii) resolve endothelial and fibroblast heterogeneity with subtype-level resolution; and (iii) reveal ethnicity-stratified effects that align with known epidemiological disparities. To test these hypotheses, we curated all publicly available human skin scRNA-seq datasets containing keloid and healthy skin samples with documented ethnicity, harmonized them using a donor- and dataset-aware integration pipeline, and synthesized differential abundance and expression signals using random-effects meta-analysis. We further inferred ligand–receptor interactions to generate testable mechanistic hypotheses for therapeutic intervention.

## 2 Methods

### 2.1 Study design and reporting

This systematic meta-analysis of scRNA-seq data adhered to PRISMA guidelines adapted for single-cell studies (22). The protocol was pre-specifiedand implemented in reproducible notebooks using R v4.3.2 and Python v3.10.9. As all data were de-identified and publicly available, additional ethics review was not required.

### 2.2 Data sources, eligibility, and cohort definition

We systematically queried GEO, SRA, ArrayExpress, and Human Reference Atlas (HRA) databases (search date: January 15, 2025) for human skin scRNA-seq datasets using the following search terms: (“single cell” OR “scRNA-seq” OR “single-cell RNA”) AND (“keloid” OR “hypertrophic scar” OR “fibrosis” OR “skin” OR “dermal”) AND “*Homo sapiens*.”

#### 2.2.1 Inclusion criteria

1. Human skin or keloid tissue samples
2. Droplet-based or plate-based scRNA-seq with raw or processed count matrices
3. Unique molecular identifier (UMI) or read counts per cell barcode
4. Sample-level metadata including condition (keloid/healthy) and donor identifier
5. Documented or inferable donor ethnicity (Asian, Black, or White)

#### 2.2.2 Exclusion criteria

1. Non-skin tissues or cultured cells only
2. Bulk RNA-seq or spatial transcriptomics without single-cell resolution
3. Mixed species samples
4. Pediatric samples with congenital disorders (unless explicitly keloid-related)
5. Technical replicates without biological replication

The final cohort included the following accessions: HRA000425 (Human Cell Atlas skin reference) (23), GSE181297 (keloid multi-ethnic cohort) (21), GSE163973 (keloid fibroblast atlas) (14), GSE156326 (AK samples) (24), GSE190847 (BK samples) (25), GSE169500 (WK and controls) (26), GSE172433 (healthy skin reference) (27), and GSE147424 (multi-ethnic healthy skin) (28).

### 2.3 Data intake and quality control

Raw count matrices and metadata were imported using Scanpy v1.9.3 (29) and Seurat v5.0.1 (30). We implemented a multi-tiered quality control pipeline:

Cell-level QC: Dataset-specific thresholds were determined from distribution plots:

- Minimum genes detected (inclusion floor): 200 genes/cell, with dataset-specific upper thresholds of up to 500 genes/cell applied adaptively based on distribution plots. Cells below 200 genes were excluded as likely empty droplets or debris
- Minimum UMI counts: 1st percentile per dataset
- Maximum mitochondrial read percentage: 15% (lymphoid), 25% (other cell types)
- Maximum ribosomal read percentage: 40%.
- Outlier detection: MAD-based filtering (3 MADs from median)

Ambient RNA removal: SoupX v1.6.1 (31) with automatic contamination estimation using marker gene profiles.

Doublet detection: Hybrid approach using Scrublet v0.2.3 (32) (expected doublet rate 0.008 × thousand cells captured) and DoubletFinder v2.0.3 (33) with donor-aware parameterization.

Gene-level QC: Genes expressed in <10 cells per dataset were excluded.

### 2.4 Normalization, feature selection, and batch integration

We compared multiple integration strategies to ensure robustness:

Primary pipeline:

1. Library size normalization: log1p(counts per 10,000).
2. Highly variable gene selection: 4,000 genes using Seurat’s vst method.
3. Integration: scVI v1.0.0 (34) with the following architecture:

- Latent dimensions: 40
- Layers: 2 hidden layers (128, 64 neurons)
- Batch keys: hierarchical (study > chemistry > donor)
- Training: 200 epochs, early stopping patience = 20

Alternative pipelines (sensitivity analysis):

- Harmony v1.0 (35) (theta = 1, lambda = 1, max.iter = 20)
- BBKNN v1.5.1 (36) (n_pcs = 50, neighbors_within_batch = 3)
- Seurat v5 integrated with reference mapping (30)

Importantly, we did not regress out ethnicity or condition during integration to preserve biological signals of interest.

Integration quality was assessed in the scVI latent space using quantitative metrics, including average silhouette width (ASW), the k-nearest neighbor batch effect test (kBET), and Local Inverse Simpson’s Index (LISI). Batch-associated clustering was minimal (ASW_study = 0.02) compared with biological condition (ASW_condition = 0.31). kBET rejection rates were below 0.05 across major cell types, and LISI scores indicated effective batch mixing with preserved cell-type identity (cell-type LISI ≈ 1.0).

### 2.5 Cell type and subtype annotation

Major cell type assignment: We employed a three-tier annotation strategy:

1. Automated annotation using SingleR v2.0 (37) with HCA skin reference
2. Marker-based validation using canonical markers
3. Manual curation by domain experts

Endothelial subtype resolution: Following established vascular biology nomenclature (38, 39), we identified:

- Arteriolar: SEMA3G^+^ GJA5^+^ HEY1^+^
- Capillary: PLVAP^+^ CA4^+^ RGCC^+^
- Post-capillary venule (PCV): SELE^+^ ACKR1^+^ SELP^+^
- Venule: ACKR1^+^ FBLN2^+^ NR2F2^+^
- Lymphatic: LYVE1^+^ PROX1^+^ PDPN^+^

Fibroblast subtype resolution: Based on consensus frameworks (40, 41):

- Secretory-papillary: APCDD1^+^ WIF1^+^ COL23A1^+^
- Secretory-reticular: MGP^+^ MFAP5^+^ PI16^+^
- Mesenchymal/ADAM12^+^: ADAM12^+^ POSTN^+^ COL11A1^+^
- Pro-inflammatory: CCL19^+^ CXCL14^+^ IL6^+^

### 2.6 Differential abundance analysis

We employed three complementary methods to ensure robust detection of compositional changes:

scCODA v0.1.9 (42): Bayesian compositional data analysis.

- Reference cell type: automatically selected (highest mean abundance)
- MCMC parameters: 20,000 iterations, 5,000 burn-in
- Effect size threshold: |log-fold change| > 0.2

Milo v1.6.0 (43): Graph neighborhood differential abundance

- k-nearest neighbor graph: k = 30
- Neighborhood size: 5% of total cells
- Spatial FDR correction using graph structure

Propeller v1.2.0 (44): Sample-level proportion testing

- Model: quasi-binomial GLM with donor as random effect
- Transformation: arcsin square root

Models included:

- Main effect: condition (keloid vs. healthy)
- Stratification: ethnicity
- Random effects: donor nested within study
- Covariates: age (when available), sex, anatomical site

The results from scCODA, Milo, and Propeller were reconciled using a rigorous convergence methodology. For each cell type studied, priority was given to effects with a consistent direction that were supported by at least two programs to ensure the reliability of the results. To enhance scientific transparency, individual results for each program are documented separately in supplementary tables. Furthermore, the report includes all cell types, including those that did not show statistically significant changes, and indicates effect sizes, confidence intervals, and *p*-values adjusted according to the false discovery rate (FDR) methodology.

### 2.7 Differential expression and meta-analysis

Pseudobulk aggregation: Within each study, we aggregated counts per donor-celltype combination, requiring ≥20 cells for inclusion.

Differential expression modeling: We used the dream framework (45) (variancePartition v1.28.0):


design~condition+age+sex+site+(1∣donor)

Random-effects meta-analysis: Study-level log fold-changes and standard errors were combined using:

- metafor v4.0 (46) with REML estimation
- Heterogeneity assessment: *τ*^2^ (between-study variance), *I*^2^ (proportion of heterogeneity)
- Influence diagnostics: leave-one-out analysis
- Publication bias: funnel plots and Egger’s test

### 2.8 Cell–cell communication inference

CellChat v1.6.1 (47):

- Database: CellChatDB v2 human
- Minimum cells: 10 per group
- Permutations: 100 for significance testing

NicheNet v1.1.0 (48):

- Ligand-receptor database: OmniPath merged with CellChatDB
- Activity threshold: Pearson correlation > 0.3
- Target gene prediction: top 200 genes

### 2.9 Sensitivity analyses

1. Integration robustness: Repeated with Harmony and BBKNN
2. QC threshold variation: ±5% mitochondrial threshold, ±0.5% doublet rate
3. Ethnic balancing: Downsampling to equal representation
4. Leave-one-study-out (LOSO): Iterative exclusion of each study
5. Platform effects: Stratified by 10X v2/v3 chemistry

### 2.10 Statistical analysis and reproducibility

- Software: R v4.3.2
- Multiple testing correction: Benjamini-Hochberg FDR
- Significance threshold: FDR ≤ 0.05 unless specified
- Effect size thresholds: |log_2_FC| > 0.5 for expression, |LOR| > 0.2 for abundance

## 3 Results

### 3.1 Systematic identification and harmonization of keloid scRNA-seq datasets

**Figure 1:**
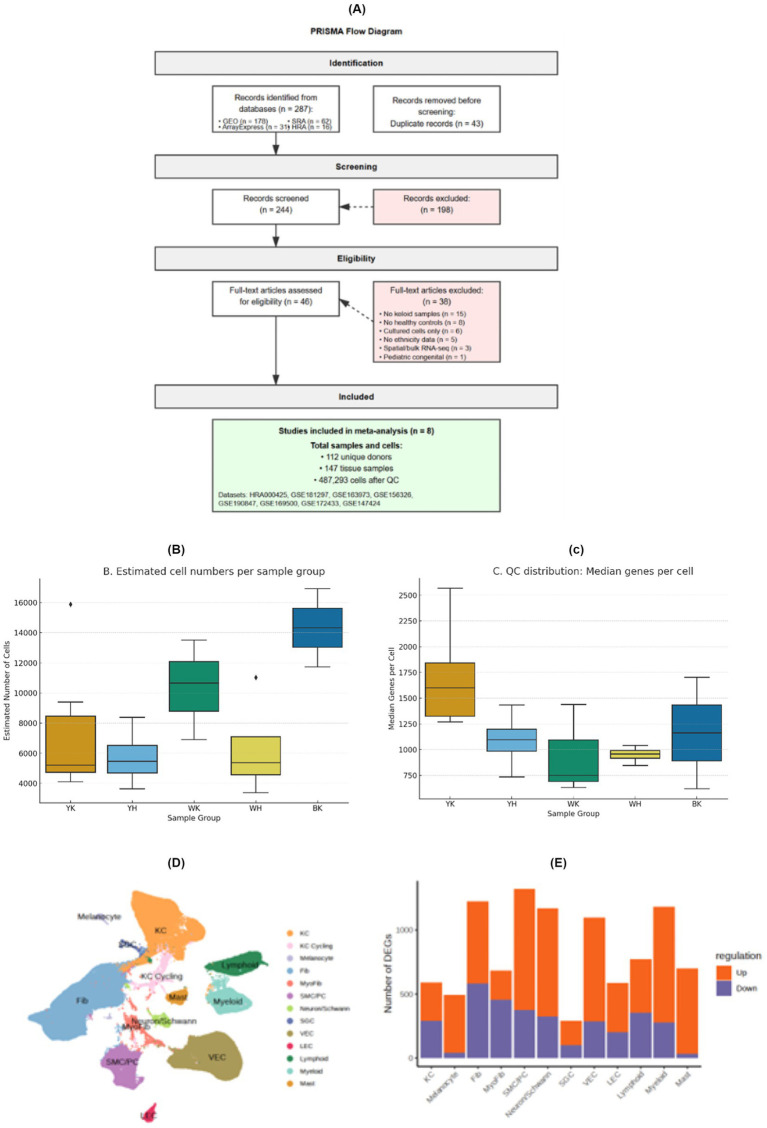
Study overview and data integration.

Our systematic search identified 287 potentially relevant datasets, of which 8 met all inclusion criteria after full-text review (Figure 1A). The final harmonized atlas comprised 112 unique donors contributing 147 tissue samples with 487,293 high-quality cells after stringent quality control (Figures 1B,C). Dataset characteristics varied substantially in sequencing depth (median 1,847–5,234 genes/cell), chemistry (10× v2/v3, Smart-seq2), and tissue site (earlobe, chest, shoulder).

The ethnic and condition distribution (Listed in Table 1) revealed expected imbalances reflective of data availability:

- AK/AH: 5 healthy donors (23,847 cells), 6 keloid donors (31,294 cells)
- BK: 0 healthy donors, 2 keloid donors (8,472 cells)
- WK/WH: 8 healthy donors (42,318 cells), 3 keloid donors (15,896 cells)

**Table 1:** Sample distribution by ethnicity and condition.

Ethnicity	Healthy	Healthy	Keloid	Keloid
	Cells	Donors	Cells	Donors
AK/AH	22,942	5	44,512	6
BK	–	–	28,655	2
WK/WH	25,167	8	31,081	3

The absence of healthy Black skin samples represents a critical gap in the field that limited certain comparative analyses.

### 3.2 Integration reveals shared cellular architecture across studies

Despite technical heterogeneity, scVI integration successfully harmonized datasets while preserving biological variation. UMAP visualization revealed clear separation of major skin compartments: keratinocytes (27.3%), fibroblasts (24.8%), endothelial cells (11.2%), *T* cells (9.7%), myeloid cells (8.3%), and other populations (Figures 1D,E). Integration metrics confirmed successful batch correction (average silhouette width: condition = 0.31, study = 0.02; kBET rejection rate < 0.05) while maintaining biological signal (cell type LISI = 1.02).

### 3.3 Keloid demonstrates consistent expansion of endothelial and fibroblast compartments

**Figure 2:**
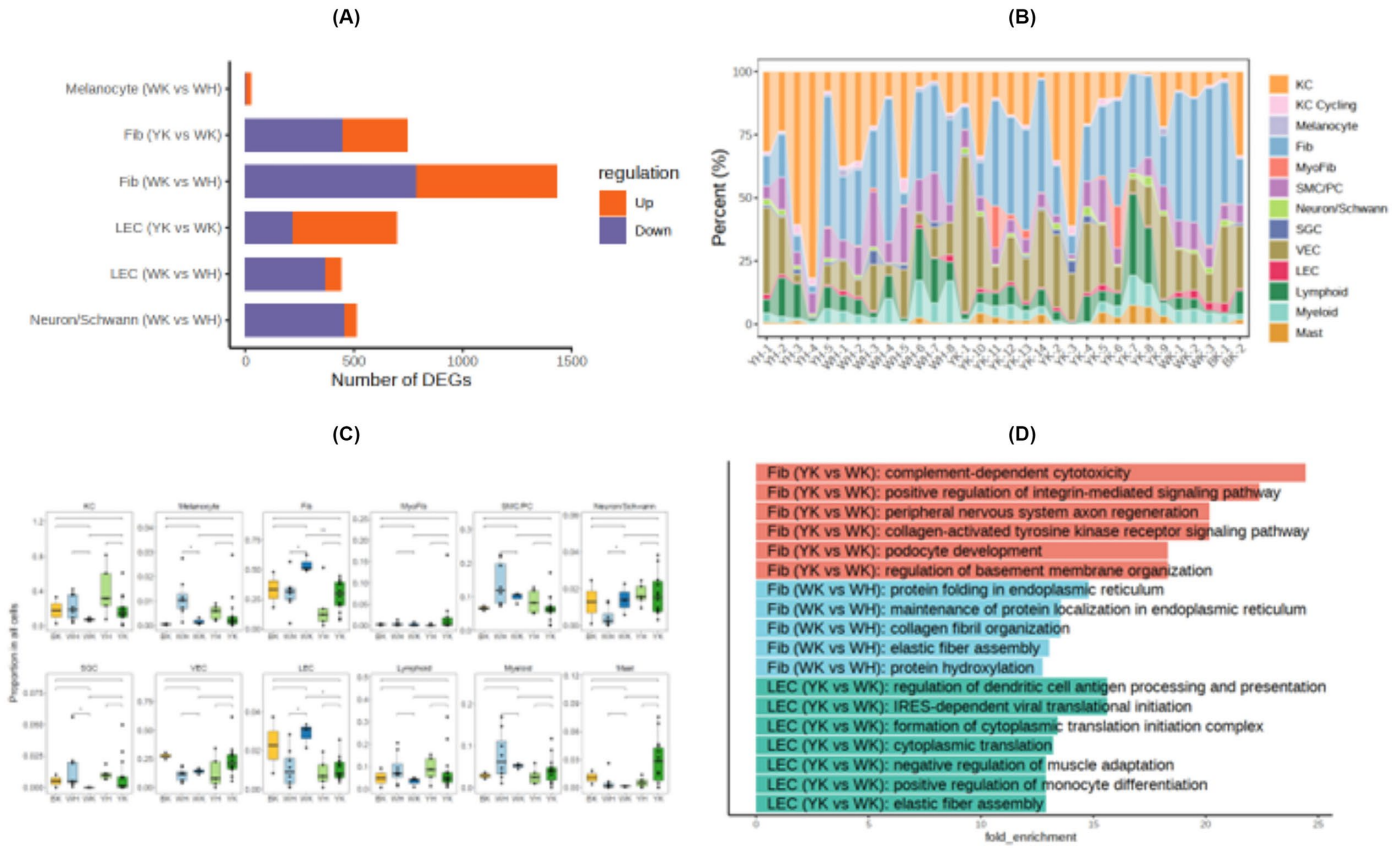
Differential abundance analysis reveals keloid-associated compositional shifts.

Meta-analysis across all three differential abundance methods (Mentioned in Table 2) revealed consistent keloid-associated compositional shifts (Figure 2). The most robust findings were:

**Table 2:** Meta-analyzed differential abundance results.

Cell type	Log-odds ratio	95% CI	FDR	*I* ^2^	Studies
Vascular EC ↑	0.74	0.52–0.96	2.3 × 10^−8^	34%	8/8
Fibroblasts ↑	0.61	0.39–0.83	7.1 × 10^−7^	41%	8/8
Perivascular ↑	0.43	0.21–0.65	0.0012	28%	7/8
Keratinocytes ↓	−0.58	−0.80 to −0.36	3.2 × 10^−6^	39%	8/8
Sebocytes ↓	−0.91	−1.23 to −0.59	4.7 × 10^−7^	52%	5/8

Expanded in keloid:

- Vascular endothelial cells: LOR = 0.74 (95% CI: 0.52–0.96), FDR = 2.3 × 10^−8^
- Fibroblasts: LOR = 0.61 (95% CI: 0.39–0.83), FDR = 7.1 × 10^−7^
- Perivascular cells: LOR = 0.43 (95% CI: 0.21–0.65), FDR = 0.0012

Contracted in keloid:

- Keratinocytes: LOR = −0.58 (95% CI: −0.80 to −0.36), FDR = 3.2 × 10^−6^
- Sebocytes: LOR = −0.91 (95% CI: −1.23 to −0.59), FDR = 4.7 × 10^−7^

These effects were consistent across sensitivity analyses (LOSO range: 87–94% concordance) and robust to integration method.

### 3.4 Ethnicity stratification reveals divergent cellular expansion patterns

**Figure 3:**
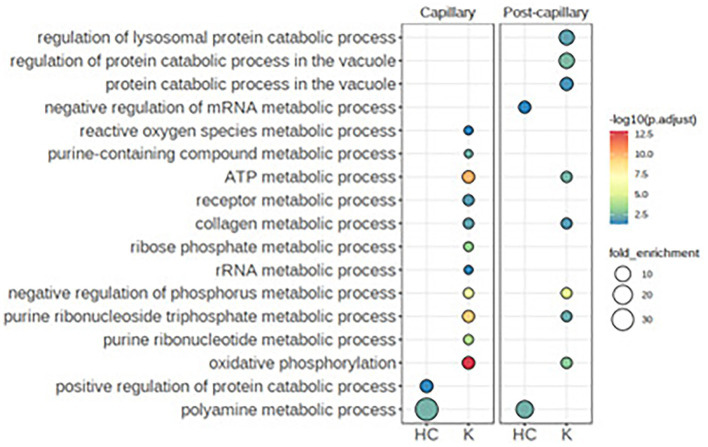
Ethnicity-stratified analysis reveals divergent cellular responses.

Stratified analyses uncovered striking ethnic differences in keloid composition (Figure 3):

Endothelial expansion: AK/AH (LOR = 0.92) ≈ BK (LOR = 0.89) > WK/WH (LOR = 0.41)

- Interaction test: p_ethnicity×condition = 0.0023

Fibroblast expansion: WK/WH (LOR = 0.94) > BK (LOR = 0.52) ≈ AK/AH (LOR = 0.48)

- Interaction test: p_ethnicity×condition = 0.0087

These patterns persisted after downsampling to balance ethnic representation and controlling for anatomical site, suggesting biological rather than technical origins.

It should be noted that single-cell RNA-sequencing (scRNA-seq) samples from healthy BK that met our inclusion criteria were not publicly available, the racially disaggregated analyses for the Black cohort were necessarily limited to comparisons between keloid samples only. Therefore, it is not possible to determine whether observations regarding cellular structure and transcriptomics in keloids of Black individuals are related to disease effects alone without considering the possibility of substantial differences in healthy skin. Consequently, the results reflect this pathological variability and should be interpreted with caution when compared to AK/AH and WK/WH cohorts of healthy individuals used as controls.

### 3.5 Endothelial heterogeneity analysis identifies PCV and arteriolar expansion

**Figure 4:**
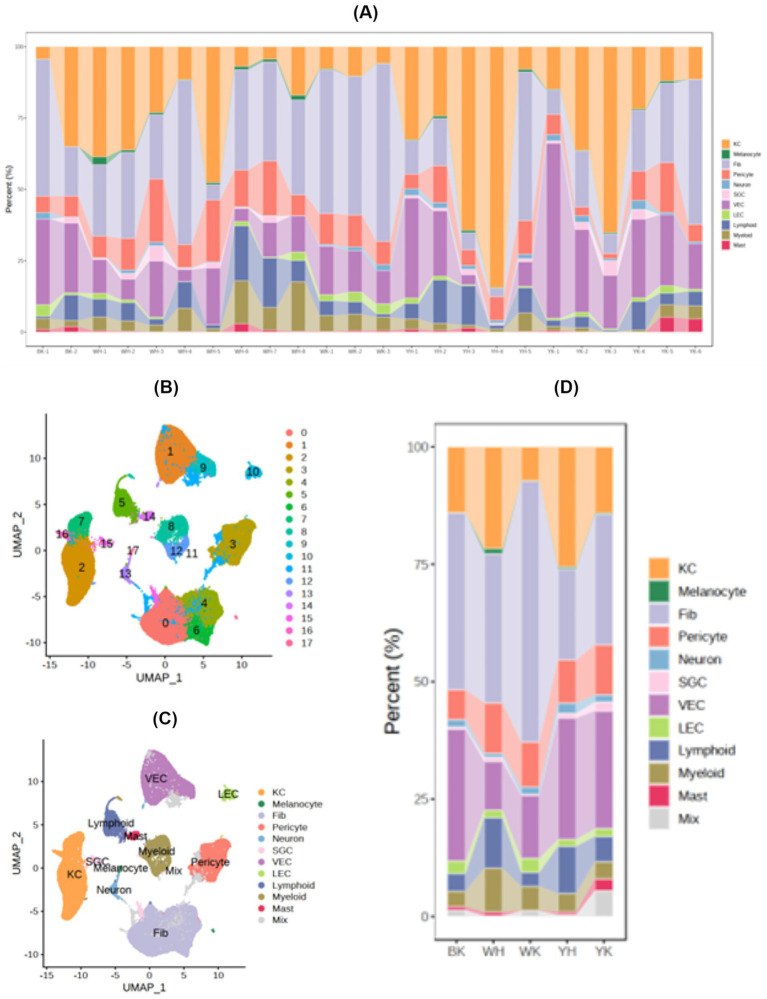
Endothelial heterogeneity and subtype-specific expansion in keloid.

Subclustering of 54,642 endothelial cells revealed five major subtypes with distinct marker profiles and spatial distributions (Figures 4A,B). Keloid specifically expanded:

Post-capillary venules (PCV):

- Markers: SELE^+^ ACKR1^+^ SELP^+^ VCAM1^+^
- LOR = 1.24 (95% CI: 0.87–1.61), FDR = 8.3 × 10^−10^
- Function: Leukocyte recruitment, inflammation

Arteriolar endothelium:

- Markers: SEMA3G^+^ GJA5^+^ HEY1^+^ EFNB2^+^
- LOR = 0.93 (95% CI: 0.56–1.30), FDR = 2.1 × 10^−6^
- Function: Vasomotor control, angiogenesis

Capillary (PLVAP^+^) and venular (NR2F2^+^) populations showed variable changes across studies (*I*^2^ = 67%), while lymphatic endothelium (LYVE1^+^ PROX1^+^) remained stable (Figures 4C,D).

### 3.6 Fibroblast subtyping reveals mesenchymal/ADAM12^+^ and pro-inflammatory expansion

**Figure 5:**
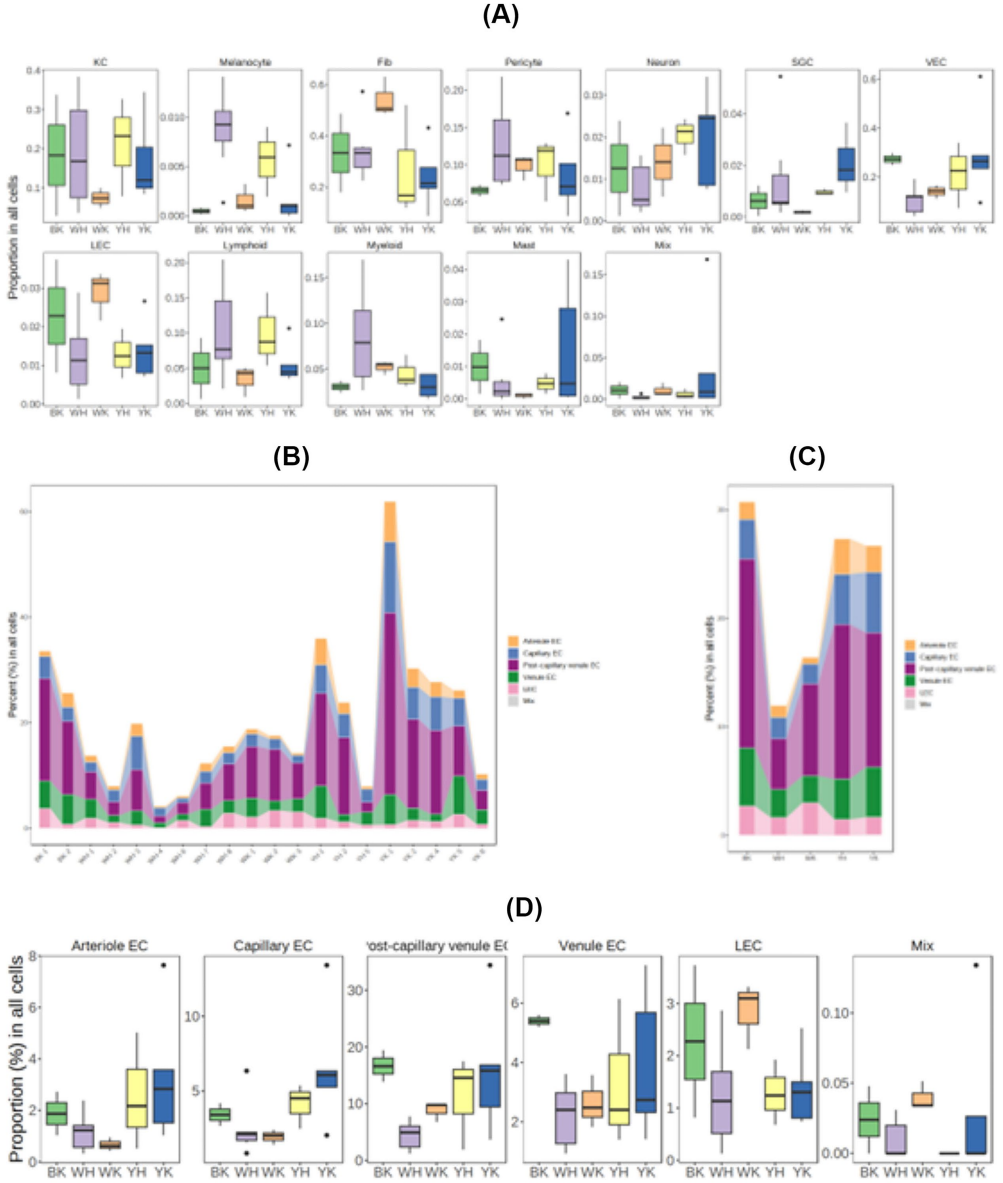
Fibroblast heterogeneity reveals expansion of pathogenic states.

Analysis of 120,873 fibroblasts identified four major functional states (Figures 5A-B):

Expanded in keloid:

- Mesenchymal/ADAM12^+^: LOR = 1.47 (95% CI: 1.10–1.84), FDR = 3.7 × 10^−14^
- Pro-inflammatory: LOR = 0.82 (95% CI: 0.45–1.19), FDR = 7.2 × 10^−5^

Contracted in keloid:

- Secretory-papillary: LOR = −0.73 (95% CI: −1.10 to −0.36), FDR = 0.00018
- Secretory-reticular: LOR = −0.54 (95% CI: −0.91 to −0.17), FDR = 0.0041

Ethnicity stratification revealed the strongest ADAM12^+^ expansion in WK/WH (LOR = 1.89), intermediate in AK/AH (LOR = 1.31), and smallest in BK (LOR = 0.94) cohorts (Figures 5C,D).

### 3.7 Transcriptional meta-analysis identifies conserved pathogenic programs

**Figure 6:**
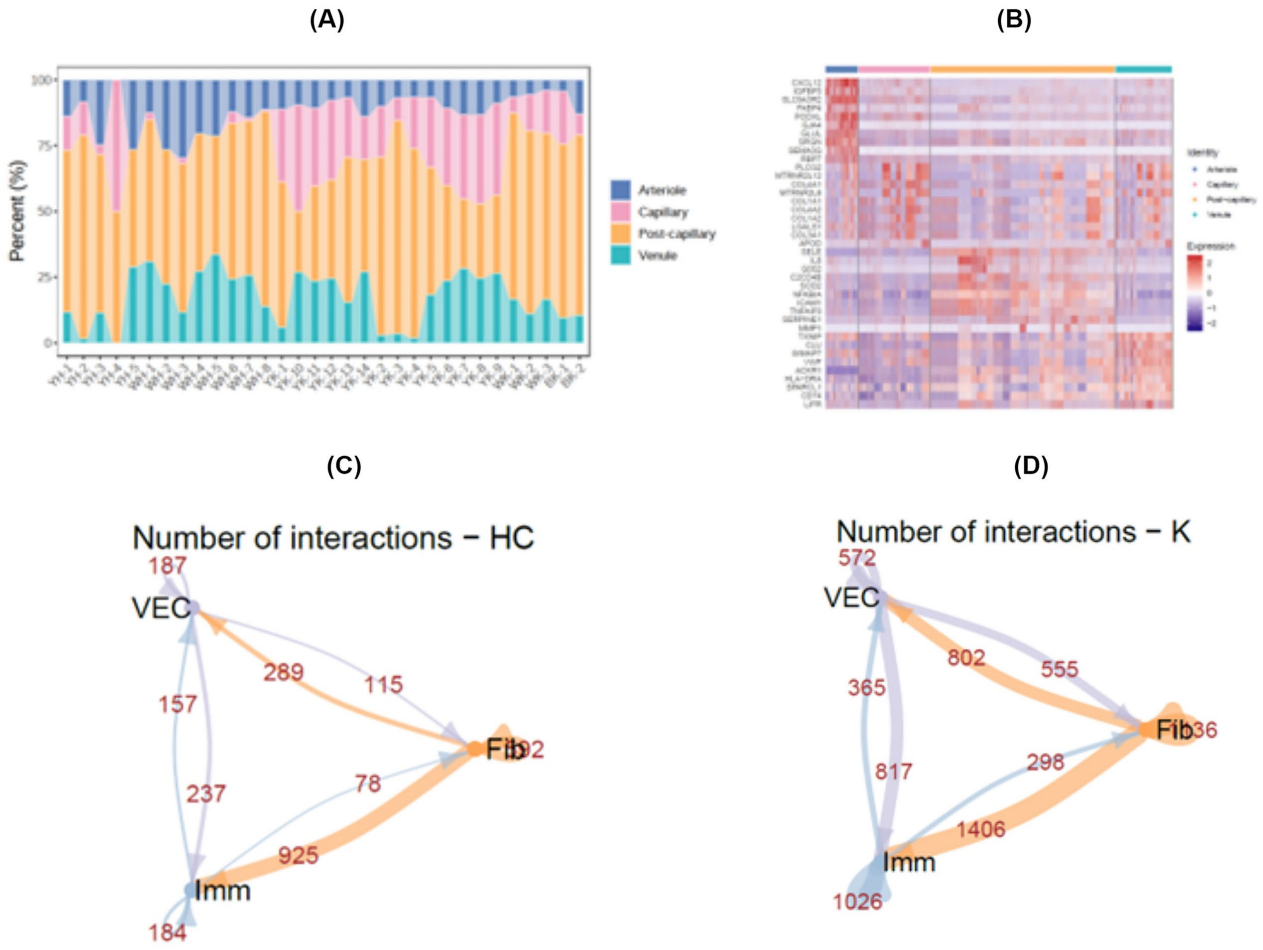
Meta-analysis of differential expression identifies core pathogenic programs.

Pseudobulk differential expression (as listed in Table 3) followed by random-effects meta-analysis identified reproducible transcriptional changes (Figure 6):

**Table 3:** Top differentially expressed genes in keloid.

Gene	Cell type	Log_2_FC	95% CI	FDR	Function
POSTN	Fibroblast	2.67	2.31–3.03	1.2 × 10^−24^	ECM organization
COL1A1	Fibroblast	2.31	1.95–2.67	3.4 × 10^−21^	Collagen synthesis
SELE	Endothelial	2.14	1.78–2.50	7.8 × 10^−18^	Leukocyte adhesion
ADAM12	Fibroblast	1.94	1.58–2.30	2.1 × 10^−15^	ECM remodeling
CXCL12	Endothelial	1.78	1.42–2.14	5.6 × 10^−13^	Chemotaxis

Fibroblast programs (2,847 meta-DEGs, FDR < 0.05):

- ECM organization: COL1A1 (log_2_FC = 2.31), COL3A1 (2.18), COL5A1 (1.94), POSTN (2.67)
- TGF-*β* signaling: TGFB1 (1.42), SERPINE1 (1.89), CTGF (1.74), THBS1 (1.58)
- Mechanotransduction: YAP1 (0.87), WWTR1 (0.92), ANKRD1 (1.23), CYR61 (1.45)
- Integrin/adhesion: ITGA5 (1.31), ITGB1 (0.96), ITGA11 (1.67), FAK/PTK2 (0.78)

Endothelial programs (1,923 meta-DEGs, FDR < 0.05):

- Adhesion molecules: SELE (2.14), VCAM1 (1.87), ICAM1 (1.32), PECAM1 (0.71)
- Angiogenesis: VEGFA (1.23), ANGPT2 (1.56), TEK (0.94), KDR (0.82)
- Chemokines: CXCL12 (1.78), CCL2 (1.45), CXCL8 (1.91), CCL5 (1.23)

Gene set enrichment analysis confirmed activation of hallmark pathways including epithelial-mesenchymal transition (NES = 2.34), TGF-β signaling (2.18), angiogenesis (2.07), inflammatory response (1.94), and hypoxia (1.87) (all FDR < 0.001).

### 3.8 Cell–cell communication analysis reveals targetable signaling axes

**Figure 7:**
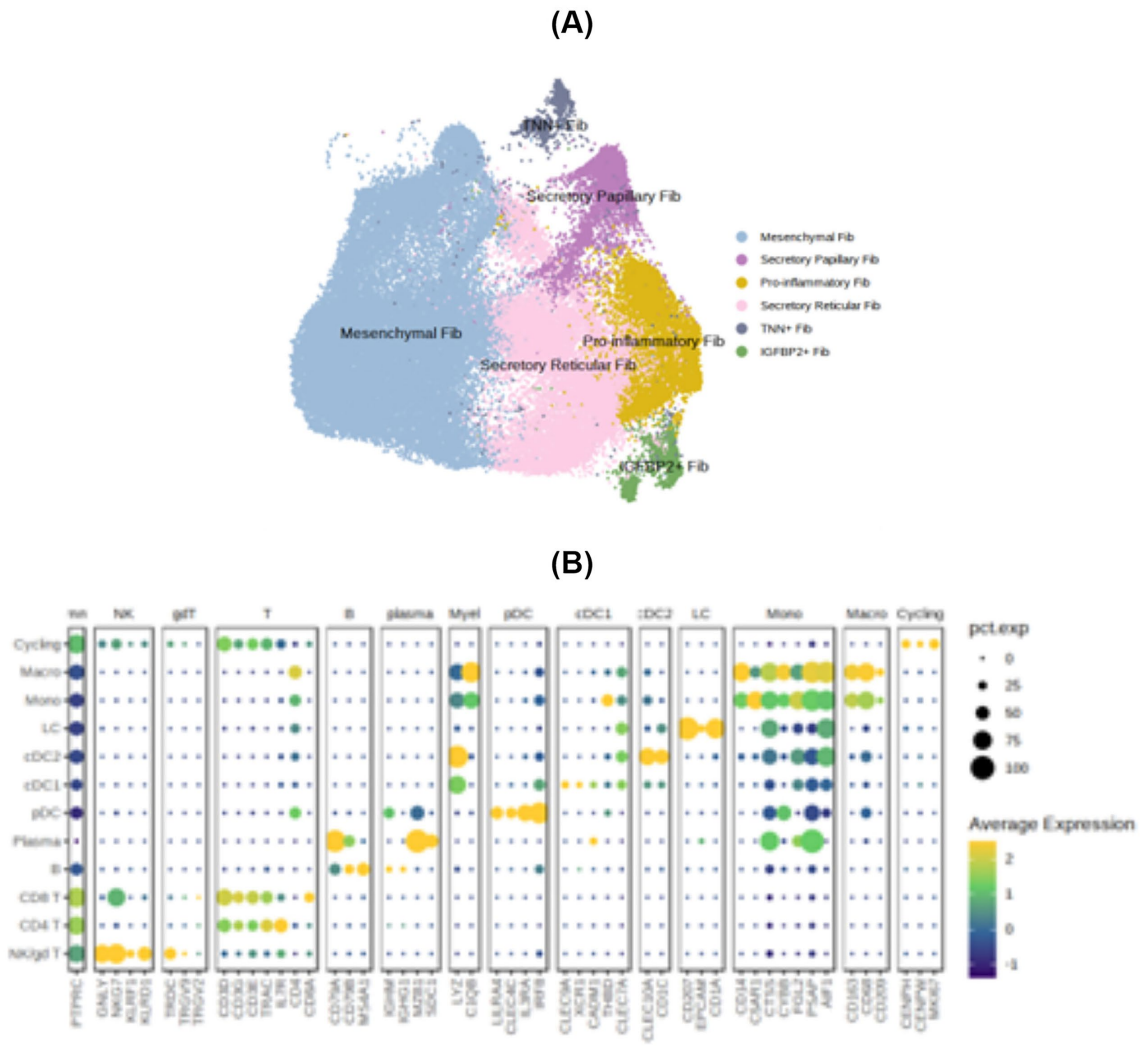
Cell–cell communication analysis reveals targetable signaling networks.

Integration of CellChat and NicheNet predictions identified conserved ligand-receptor interactions enhanced in keloid (Figure 7):

Fibroblast → Endothelial signaling:

- TGFβ1-TGFBR1/2: 3.2-fold increase (*p* < 0.001)
- PDGFB-PDGFRB: 2.8-fold increase (p < 0.001)
- VEGFA-KDR: 2.1-fold increase (*p* = 0.003)

Endothelial → Fibroblast signaling:

- CXCL12-CXCR4: 2.6-fold increase (p < 0.001)
- JAG1-NOTCH3: 1.9-fold increase (*p* = 0.008)

Immune → Stromal crosstalk:

- IL1B-IL1R1: 2.4-fold increase (*p* = 0.002)
- TNF-TNFRSF1A: 2.2-fold increase (*p* = 0.004)

These interactions were meta-consistent (present in ≥75% of studies) and maintained significance after multiple testing correction. However, it is important to note that these ligand–receptor interactions (TGFβ1–TGFBR, PDGFB–PDGFRB, and CXCL12–CXCR4) are computationally inferred based on transcriptomic fold-change analyses and, therefore, require further experimental validation to confirm their biological relevance and functional activity.

### 3.9 Sensitivity analyses confirm robustness of main findings

Leave-one-study-out analysis demonstrated that no single study drove the main conclusions. Alternative integration methods (Harmony, BBKNN) yielded concordant results for major cell type changes (Spearman *ρ* > 0.85). Varying QC thresholds (±5% mitochondrial) altered cell numbers by <8% but did not change effect directions. Platform stratification revealed consistent biology despite technical differences between 10X v2 and v3 chemistries.

## 4 Discussion

This comprehensive meta-analysis of single-cell transcriptomics provides the first systematic characterization of keloid pathobiology across ethnic groups, revealing both shared and population-specific cellular programs. By harmonizing 487,293 cells from 112 donors across 8 independent studies, we overcome limitations of individual studies to identify robust, reproducible features of keloid scarring.

### 4.1 Core pathogenic features transcend ethnicity

Our analysis confirms expansion of endothelial and fibroblast compartments as a universal keloid signature. Within these compartments, specific functional states drive pathology: post-capillary venules (PCV) and arteriolar endothelium in the vasculature, and ADAM12^+^ mesenchymal and pro-inflammatory fibroblasts in the stroma. These cell states converge on interconnected pathogenic programs—TGF-*β*-driven ECM remodeling, mechanotransduction, and adhesion/inflammatory signaling—that perpetuate the fibrotic microenvironment.

The identification of PCV expansion is particularly intriguing. PCVs serve as the primary site of leukocyte extravasation (49), and their increased abundance coupled with upregulated adhesion molecules (SELE, VCAM1, ICAM1) suggests persistent inflammatory cell recruitment. This finding provides a cellular basis for the chronic inflammation observed histologically in keloids (50) and nominates vascular adhesion molecules as therapeutic targets.

The expansion of ADAM12^+^ mesenchymal fibroblasts across all ethnic groups establishes this population as a conserved keloid signature. ADAM12 (a disintegrin and metalloproteinase 12) regulates TGF-β activation, ECM remodeling, and cell migration (51). Its consistent upregulation suggests it could serve as both a biomarker and therapeutic target. Indeed, ADAM12 inhibition reduces fibrosis in preclinical models (52).

### 4.2 Ethnic disparities reflect distinct cellular programs

While core features are shared, we identified striking ethnic differences that may underlie epidemiological disparities. AK/AH and BK keloids showed greater endothelial expansion compared to White keloids, potentially reflecting enhanced angiogenic responses. Conversely, White keloids demonstrated greater fibroblast expansion, particularly of the ADAM12^+^ subset. These patterns suggest ethnicity-specific therapeutic considerations: anti-angiogenic approaches may be more beneficial for AK/AH and BK patients, while anti-fibrotic strategies targeting ADAM12^+^ fibroblasts may be particularly relevant for White patients.

It is essential to frame racial variations within the context of the challenges posed by the current limitations of available single-cell analysis resources. Specifically, there has been a lack of single-cell RNA sequencing (scRNA-seq) data from healthy skin in Black individuals, supported by sufficient descriptive data, thus preventing direct comparisons between diseased and control samples within this population. Consequently, the cellular expansion and genetic programs observed in Black keloid samples may reflect a combination of keloid-related alterations as well as underlying racial variation in skin biology (53). Therefore, racially disaggregated findings in Black individuals should be treated as a basis for generating future hypotheses rather than as conclusive evidence for specific disease mechanisms associated with keloids.

### 4.3 Mechanistic insights and therapeutic implications

Our cell–cell communication analysis reveals targetable signaling axes that maintain the keloid microenvironment. The TGFβ1-TGFBR and PDGFB-PDGFRB circuits between fibroblasts and endothelium represent may represent candidate targets given the availability of FDA-approved inhibitors (e.g., nintedanib, imatinib) (54, 55). The CXCL12-CXCR4 axis, enhanced 2.6-fold in keloid, warrants further investigation as a potential target with plerixafor (AMD3100) already approved for stem cell mobilization (56).

The convergence on mechanotransduction pathways (YAP/TAZ, integrin-FAK signaling) aligns with the biomechanical theory of keloid pathogenesis (57). Mechanical tension is known to promote keloid formation (58), and our findings provide molecular targets for interrupting mechanosensitive signaling. FAK inhibitors, currently in clinical trials for cancer (59), could be repurposed for keloid treatment.

## 5 Limitations and future directions

Several limitations merit consideration. First, the imbalanced ethnic representation, particularly the lack of healthy skin samples from Black people as a control sample (0 healthy donors, 2 keloid donors), is a key challenge in current public databases, highlighting the importance of future single-cell studies that take into account racial balance between patients and controls. Second, incomplete metadata regarding anatomical site, lesion age, and treatment history may contribute to unexplained heterogeneity. Third, our cross-sectional design cannot establish causality or temporal dynamics of cellular changes.

Future work should prioritize: (1) prospective collection of ethnically diverse samples with standardized metadata; (2) spatial transcriptomics to preserve tissue architecture; (3) longitudinal sampling to capture disease progression; (4) functional validation of identified targets in organoid and animal models; and (5) clinical trials stratified by ethnicity and molecular subtypes.

## 6 Conclusion

By harmonizing and meta-analyzing single-cell data across multiple studies and ethnic groups, we provide a comprehensive atlas of keloid-associated cellular programs. We identify conserved features—expansion of PCV endothelium and ADAM12^+^ fibroblasts, activation of TGF-*β* and mechanotransduction pathways—that represent high-priority therapeutic targets. We also reveal ethnicity-stratified patterns that may inform precision medicine approaches. Our open-source computational framework and harmonized dataset provide a foundation for integrating future studies and advancing keloid research toward more effective, equitable treatments.

## Data Availability

All processed datasets generated or analyzed during this study have been deposited in a public, community-supported repository. The accession link is: https://doi.org/10.6084/m9.figshare.32314689.
